# Advances in Proteomic and Metabolomic Profiling of Neurodegenerative Diseases

**DOI:** 10.3389/fneur.2021.792227

**Published:** 2022-01-31

**Authors:** Artur Schumacher-Schuh, Andrei Bieger, Wyllians V. Borelli, Makayla K. Portley, Paula Saffie Awad, Sara Bandres-Ciga

**Affiliations:** ^1^Departamento de Farmacologia, Universidade Federal do Rio Grande do Sul, Porto Alegre, Brazil; ^2^Serviço de Neurologia, Hospital de Clínicas de Porto Alegre, Porto Alegre, Brazil; ^3^Department of Biochemistry, Universidade Federal do Rio Grande do Sul, Porto Alegre, Brazil; ^4^Neurodegenerative Disorders Clinic, National Institute of Neurological Disorders and Stroke, National Institutes of Health, Bethesda, MD, United States; ^5^Movement Disorders Clinic, Centro de Trastornos de Movimiento (CETRAM), Santiago, Chile; ^6^Laboratory of Neurogenetics, Molecular Genetics Section, National Institute on Aging, National Institutes of Health, Bethesda, MD, United States

**Keywords:** proteomics, metabolomics, Alzheimer's disease, AD, Parkinson's disease, PD, amyotrophic lateral sclerosis, ALS

## Abstract

Proteomics and metabolomics are two emerging fields that hold promise to shine light on the molecular mechanisms causing neurodegenerative diseases. Research in this area may reveal and quantify specific metabolites and proteins that can be targeted by therapeutic interventions intended at halting or reversing the neurodegenerative process. This review aims at providing a general overview on the current status of proteomic and metabolomic profiling in neurodegenerative diseases. We focus on the most common neurodegenerative disorders, including Alzheimer's disease, Parkinson's disease, and amyotrophic lateral sclerosis. We discuss the relevance of state-of-the-art metabolomics and proteomics approaches and their potential for biomarker discovery. We critically review advancements made so far, highlighting how metabolomics and proteomics may have a significant impact in future therapeutic and biomarker development. Finally, we further outline technologies used so far as well as challenges and limitations, placing the current information in a future-facing context.

## Introduction

The diagnosis of neurodegenerative diseases mostly relies on clinical presentation, sometimes aided by neuroimaging interpretation. A major caveat is that these conditions are clinically heterogeneous and often reflect a spectrum of neurodegenerative processes with intra- and inter-patient variation and complex pathologies. A growing body of evidence suggests that the neurobiological basis of distinct pathologies may share the same clinical phenotype. Thus, an accurate diagnosis is even more challenging due to the inability to access brain tissue *in vivo* and to the fact that peripheral tissues often do not reflect early stages of brain pathology.

Despite progress being made in the fields of genetics and transcriptomics (1), additional evidence of biological patterns indicative of the presence of pathology is needed. Proteomics (large-scale study of proteins) and metabolomics (large-scale study of small molecules, commonly known as metabolites) are two growing and emerging fields holding promise to investigate changes within cells, biofluids, or tissues that could give us further insight into the disease process.

The progress made in “omics” research combining multiple layers of high-throughput sources of biological information is critical in our continued effort toward a better understanding of neurodegenerative conditions.

**Figure 1:**
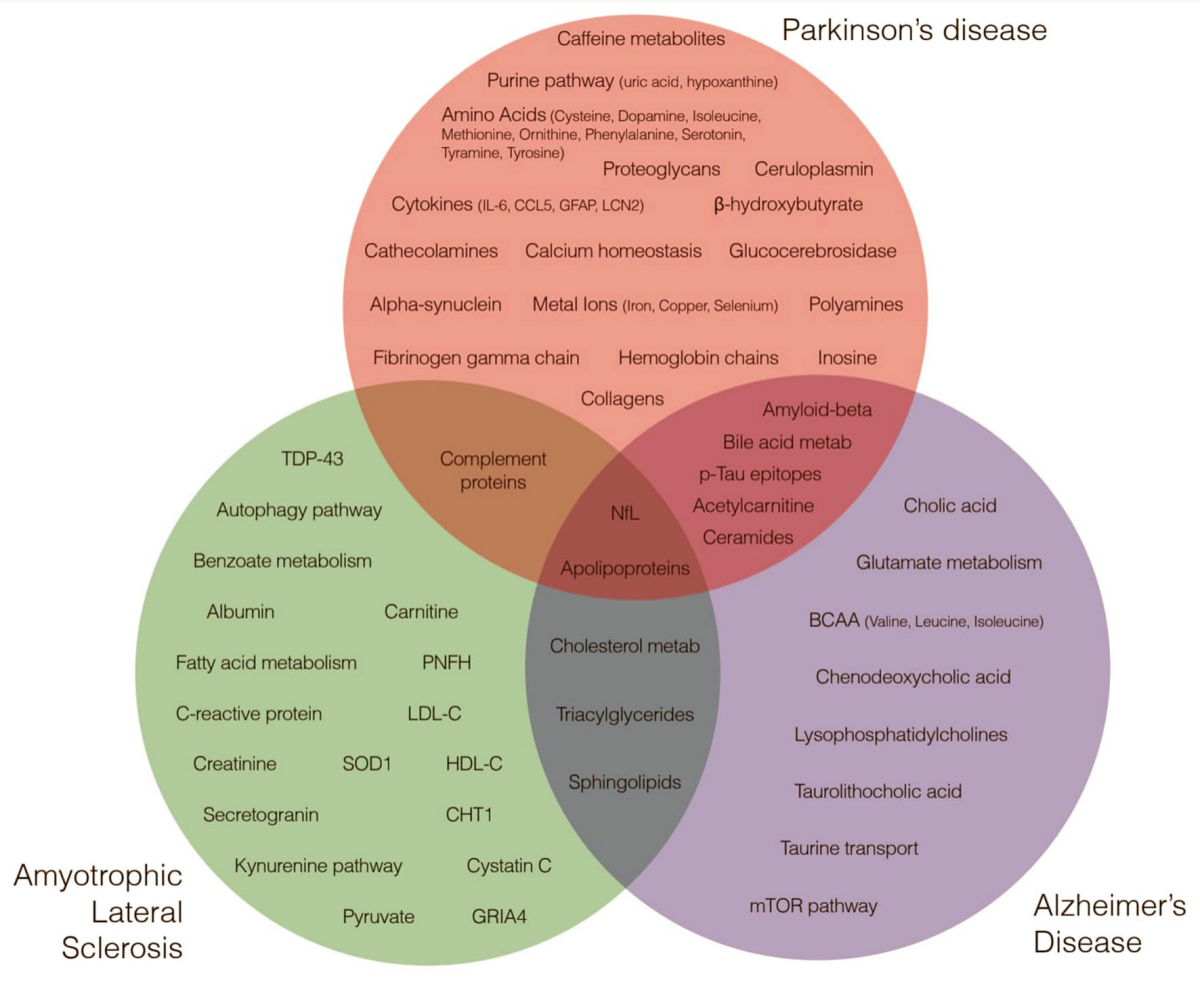
Metabolites and Proteins across Neurodegenerative Diseases. Green, red, and blue circles highlight major metabolites and proteins nominated by research studies as implicated in amyotrophic lateral sclerosis, Parkinson's disease and Alzheimer's disease etiology.

Both metabolites and proteins reflect the physiological and pathological status of an individual. Profiling these data types could be useful to identify sensitive and effective markers for early disease detection and potentially effective therapeutic interventions. Research in the context of neurodegenerative diseases may reveal and quantify specific metabolites and proteins playing a role on cellular pathways suitable for therapeutic interventions aimed at halting or reversing the neurodegenerative process (Figure 1).

## Metabolomic Studies

Our metabolome represents the interaction between genome and environment. A metabolomic intermediate can link genetics with environmental factors to influence clinical outcomes and drug responses (2). It includes both endogenous metabolites that are naturally produced in an organism such as amino acids, organic acids, nucleic acids, fatty acids, amines, sugars, vitamins, co-factors, and pigments, as well as exogenous chemicals such as drugs, environmental contaminants, food additives, toxins and other xenobiotics that are not naturally produced by an organism (3). Current technologies allow rapid screening of metabolites in an unprecedented manner, including high-resolution methods such as nuclear magnetic resonance spectroscopy and mass spectrometry (4). The advancement of these technologies has facilitated and promoted the creation of freely available metabolome databases such as the Human Metabolome Database or HMDB (containing detailed information about over 40,000 metabolites that have already been identified or are likely to be found in the human body) (5), the Urine Metabolome Database (https://urinemetabolome.ca/), the Cerebrospinal Fluid Metabolome Database (https://csfmetabolome.ca/) and the Serum Metabolome Database (https://serummetabolome.ca/), among others.

As metabolites usually constitute the final stage of biological processes, studying them simultaneously may be particularly beneficial in multifactorial neurodegenerative conditions, where dysfunction in multiple processes play a role. Ultimately, several studies have used metabolomics in different biological substrates to search for less invasive and more accurate biomarkers across many diseases, and small molecules show promise in this regard. Despite the fact that these studies provide exciting new findings, they frequently use small sample sizes, carry sources of bias or variable selection criteria, and lack uniformity in the collection and analysis of samples, precluding their immediate use in the clinic.

## Proteomic Studies

It is now widely known that protein toxicity can arise from the accumulation, mis-localization, post-translational modification such as phosphorylation, ubiquitination, methylation, acetylation, glycosylation, oxidation, and nitrosylation or multimerization of proteins which in turn can lead to neurodegeneration through mechanisms still being unveiled (6, 7). These changes result in catastrophic downstream processes, from inflammation to cell death. Unraveling the structure and function of each protein in our proteome and the complexities of protein–protein interactions is crucial for developing disease-modifying therapies and reliable diagnostic techniques as we move forward to a deeper understanding of these diseases. Current technologies to screen our proteome include mass spectrometry and protein microarrays. In the context of neurodegenerative diseases, the number of proteomic studies keep growing.

This review aims at providing a general overview on the current status of proteomic and metabolomic profiling by focusing on the most common neurodegenerative diseases, including Alzheimer's disease (AD), Parkinson's disease (PD), and amyotrophic lateral sclerosis (ALS). We critically review advancements made so far, highlighting how metabolomics and proteomics will likely have a significant impact in the future. We further outline technologies used so far, challenges and limitations and place the current information in a future facing context.

## Technologies Used to Study Metabolomics and Proteomics

### Metabolomics

Metabolomics holds immense promise in the realm of personalized medicine for identifying various biological endpoints for analysis and could therefore be utilized for diagnosis, monitoring, and identifying novel pathways and systems involved in a disease as well as therapeutic targets. While use and sophistication of these tools are ever evolving, there remain limitations. Metabolomics communicate data that is highly sensitive, responding to the genetics, gut microbiota, and environment of an individual. Confounders and overall study design need to be carefully considered to reduce interindividual variation. Additionally, though many metabolites are nominated through research, each must be validated by functional studies. In fact, many metabolites that are indicated are not replicable or fail to be validated. The use of small data sets is a key limitation in metabolomics and combining datasets shows a lot of promise in resolving some of these false positives.

Metabolomic technologies provide rich data for analyses. Mass spectrometry (MS) measures mass-to-charge ratio of ions, identifying and quantifying molecules of a sample. It is often coupled with gas chromatography (GC), ion chromatography (IC), or liquid chromatography (LC) and is a high-throughput process. Nuclear magnetic resonance (NMR) spectroscopy, a non-destructive analysis, can be used to study intact tissues and living samples in addition to biofluid or tissue extracts and can be recorded from multiple nuclei (^1^H, ^13^C, ^15^N, and ^31^P), the correlation of which can be achieved through multidimensional NMR. NMR is, however, less sensitive and selective than LC-MS and GC-MS (8).

### Proteomics

Proteomics holds many of the strengths and limitations of metabolomics, producing high-throughput data that is vulnerable to many confounders. While proteomics may not be able to explain disease development fully, it provides key insights into potential biomarkers. As with metabolomics, combining data sets to increase reliability and reproducibility of results is essential and can identify potential tools for diagnosis (9). MS is also used frequently in proteomics, with LC-MS commonly used for complex samples and matrix assisted laser desorption ionization-time-MS (MALDI-MS) used for simple protein samples (10). Advances in labeling techniques to improve identification of least abundant sample proteins have been vital to creating a full profile of conditions.

Isotope-coded affinity tag (ICAT) is also known for detection of proteins at low expression levels but cannot detect proteins without cysteine residues or acidic proteins, leaving an incomplete picture of the proteome (11). Other technologies, such as stable isotope labeling with amino acids in cell culture (SILAC), involve directly isotope labeling cells to reveal differential expression patterns, but cannot be used in tissue sample, real-time or *in vivo* measurements.

No single technology can provide a complete picture of the proteome or the metabolome, but advances have shown the complementary strengths of different technologies. Additionally, the need for combined datasets has been highlighted in both proteomics and metabolomics as a way to create more robust data and analyses.

## Alzheimer's Disease

Alzheimer's Disease (AD) is the most common form of neurodegeneration. It is a significant and growing burden, with prevalence expected to triple worldwide by 2050 (12). Since its description in the start of the twentieth century, the diagnosis of AD has evolved, and it now incorporates biological and clinical features (13). Preclinical stages of the disease begin with neuronal, glial, and vascular dysfunctions associated with amyloid beta (Aβ) accumulation in senile plaques, hyperphosphorylated tau (p-tau) aggregation in neurofibrillary tangles, and ultimately cell death (13). Numerous other biochemical processes are disrupted in AD, including lipid synthesis, mitochondrial function, inflammation, and neurotransmitter metabolism. Following these paths allowed for the development of fluid biomarkers with enough precision to be used in diagnostic criteria and to facilitate research of new therapeutic targets (14). However, the present biomarkers entail invasive procedures (lumbar puncture to retrieve CSF) or are expensive (PET scans), and while accurate, these could be substantially improved. Additionally, as Aβ is increasingly recognized as insufficient to cause neurodegeneration on its own, the field is constantly pursuing new hypotheses to explain the starting point of the disease, as well as new diagnostic and prognostic biomarkers, preferably from less invasive matrices. Metabolomics and proteomics hold great promise for improving our understanding of AD mechanisms, identifying novel biomarkers, monitoring therapy effectiveness, and developing innovative treatments.

### Metabolomics

With most studies focused on protein alterations, metabolome-driven studies are helping to shed new light into other classes of molecules putatively involved in AD pathogenesis. Interestingly, Xu et al. (15) used an unsupervised learning approach to uncover lipid, protein, and gene expression levels in a cohort of ~600 individuals and demonstrated integrated networks of lipids and proteins associated with AD, particularly those implicated in lipid metabolism and innate immunity. Furthermore, Clark et al. (16) have recently conducted multi-omics analyses in the CSF of 120 elderly participants with normal cognition, mild cognitive impairment (MCI), and mild dementia. Their results proposed new pathways associated with AD, such as hemostasis, immune response, and extracellular matrix signaling. The authors describe a combination of molecules that predict cognitive decline and dementia, as well as varying multi-omics signatures associated with neuronal injury, amyloid and tau pathology (15). In another recent study, Huo et al. (17) used a targeted multi-omics strategy to evaluate blood and brain samples from two distinct longitudinal cohorts. The authors describe a relationship between three serum acylcarnitine levels and a lower risk of AD and cognitive decline. They report a total of 13 serum metabolites that predict cognitive decline, and 28 brain metabolites related to neuropathological measurements. Aside from suggesting potential blood biomarkers for AD, this study found no substantial overlap between blood and brain signatures, implying separate metabolic dysfunction in both tissues.

Considering the expected disruption in the blood-brain barrier with aging and that AD elicits increased metabolite exchange between these tissues, blood-based biomarkers have become an attractive approach (18, 19). Xicota et al. (20) performed a transcriptomic, metabolomic, and lipidomic analysis of plasma from 48 individuals with and without amyloid deposition on PET scans. They identified several transcripts and metabolites associated with inflammation and fatty acid metabolism that distinguished those groups, implying a molecular signature for amyloid deposition that could be obtained via peripheral blood collection, a less expensive and less invasive procedure than PET scan or CSF analysis, respectively (13). Notably, Niedzwiecki et al. (21) performed a high-resolution screening in blood and CSF from two cohorts and identified three different compounds in the blood associated with CSF AD biomarkers. The study also reinforced the technique's potential to unveil novel pathways since one of the identified molecules was an unknown halogenated compound.

Several interesting compounds related to AD have been identified by lipidomic research, including sphingolipids, phospholipids, and ceramides (22). Ceramide levels in the serum and cerebrospinal fluid have been linked to memory impairment, hippocampal volume loss, and progression of AD (23, 24). Barupal et al. (25) recently examined coregulated sets of blood lipids in a total of 806 individuals from the ADNI cohort (http://adni.loni.usc.edu/) and identified seven sets of lipids linked to AD and four to cognitive deterioration. Among the screened metabolites, glucosylceramides, lysophosphatidylcholines, and triacylglycerides were shown to be associated with CSF Aβ, while sphingomyelins and ceramides were found to be associated with CSF total tau and brain atrophy. Similarly, Teitsdottir et al. (26) examined 60 individuals from an Icelandic memory clinic and reported that ceramide C18 was a distinguishing factor between AD patients and controls, a finding previously described elsewhere. Ceramides are second messengers that are created via sphingomyelin breakdown or synthesis from serine and palmitate. These compounds serve various cellular roles, most notably controlling proliferation, senescence, and cell death (27). In AD, ceramides have been previously correlated with insulin resistance and atherosclerosis (28, 29). Additionally, they are involved in the stability of the amyloid precursor protein cleavage enzyme beta-secretase 1 and the generation of Aβ (30). Interestingly, compounds that reduce ceramide levels are being investigated as possible therapeutic targets *in vitro* (31).

A recent study targeted metabolomic profiling of primary and secondary bile salts in the ADNI cohort and compared these results to the traditional AD CSF and neuroimaging biomarkers (32). The authors reported distinct bile salts signatures associated with CSF Aβ 1–42, CSF p-tau, and brain atrophy. Unsurprisingly, MahmoudianDehkordi et al. (33) conducted a similar approach in the same cohort, but this time employing clinical data. The authors identified that AD patients had a lower serum cholic acid content compared to controls (33). Additionally, they described bile acid patterns related to cognitive decline, implying changes in the gut microbiome. These findings were corroborated by Baloni et al. (34), who performed transcriptome analysis on 2,114 brain tissues and reported that genes involved in taurine transport, cholesterol metabolism, and bile acid production were differentially expressed in people with AD. Bile salts are produced from cholesterol and are involved in lipid breakdown and vitamin absorption. They are digested by the gut microbiota, which has been linked to neurodegeneration (35). It is worth highlighting that certain bile acids, such as taurolithocholic acid and chenodeoxycholic acid, are suspected to have harmful and protective effects on the nervous system, respectively (34).

Through metabolomics investigations, other intriguing chemicals identified in AD are branched-chain amino acids (BCAA), composed of an aliphatic side chain connected by a branch. In humans, the three essential BCAAs for protein synthesis are valine, leucine, and isoleucine. Tynkkynen et al. (36) used nuclear magnetic resonance and mass spectrometry metabolomics to examine 22,623 individuals from 11 separate cohorts with over 2,000 incident cases of AD. Each of the three BCAAs were associated with a decreased risk of dementia and AD. After accounting for the potential confounding effect of nutritional deficiency in people in pre-dementia stages, the authors observed that albumin levels were not associated with dementia in their data. However, they described an inverse association between creatinine and AD, implying that physical inactivity and the resulting loss of muscle mass may play a role. Under these findings, Toledo et al. (37) reported slower cognitive decline and less brain atrophy associated with increased levels of valine in the ADNI cohort, and suggested that alteration in BCAA degradation was associated with AD in a pathway analysis derived from mass spectrometry (37). Another possible explanation for these findings is that BCAAs affect glutamate metabolism, resulting in calcium dysregulation and decreased plasticity as well as their effect on tau phosphorylation via the mTOR pathway (38, 39).

### Proteomics

Our understanding of the Aβ and tau pathologies and the subsequent identification of CSF and neuroimaging biomarkers enabled the designation of AD as a biological entity, leading to a redefinition of the disease. AD can now be defined as a biological entity with a decrease in the CSF Aβ and an increase in p-tau, which opened new avenues for diagnosis, prognosis, and therapeutics (9, 40, 41). A recent systematic review and meta-analysis approached the diagnostic accuracy of these molecules in the blood, which requires less invasive and less expensive procedures to be acquired (42). Plasma Aβ1–42/Aβ1–40 ratio and p-tau significantly correlated with Aβ accumulation detected by PET scans, and the latter also predicted AD progression. A mass spectrometry assay to detect Aβ1–42/Aβ1–40 ratio demonstrated strong diagnostic performance to be commercially available (43). Other studies further depicted these known biomarkers while analyzing p-tau isoforms based on their specific phosphorylation patterns and suggested improving accuracy, especially in the early stages of the disease (44–46). Neurofilament Light Chain (NfL) has been extensively studied in AD as a marker of neurodegeneration, with a great potential for clinical practice despite its low specificity for this disease. A recent network meta-analysis provided strong evidence for its clinical use to identify early alterations in AD, among other neurodegenerative diseases (47). Since astrocytic reactions have been recognized as a major driver of AD pathology, the glial fibrillary acidic protein (GFAP) emerges as a potential biomarker (48). Higher GFAP seems to be an early marker of cognitive decline, with predictive value for the development of dementia and disease progression. However, it does not seem to be a specific marker of AD and further studies are needed to define its clinical performance (49).

There is a broad number of proteomics studies conducted in AD, often including methodological heterogeneity and distinct inclusion criteria, which prevents the generalizability of the results. Moreover, showing association does not imply causation, and further functional studies are warranted. Summarizing the current literature, Pedrero-Prieto et al. (50) conducted a systematic review and built a database of all CSF proteins differentially expressed between 2,022 AD patients and 2,562 controls from 47 studies, suggesting a panel of 27 proteins and 21 peptides as a potential tool aiding AD diagnosis (50). Similarly, Bai et al. (51) performed a meta-analysis of seven datasets generated by three different groups that used an ultra-deep proteome coverage platform. Their results point to several differentially expressed proteins associated with various cell types, like neurons, glial and epithelial cells.

Despite our knowledge about the amyloid and tau pathology, the complete picture of AD pathophysiology remains elusive, and novel proteomic biomarkers are needed. The application of next-generation proteomic techniques, such as improvements in sample processing and high-throughput mass spectrometry, in conjunction with large cohorts of well-characterized individuals, hold the prospect of achieving these goals (51). For example, Tijms et al. (52) used proteomic data from two independent cohorts in a data-driven clustering analysis. The authors found three specific protein profiles—hyperplasticity, innate immune activation, and blood-brain barrier dysfunction, suggesting different pathophysiologic subtypes for AD (52).

Furthermore, several studies suggest that the use of sensitive and precise panels of blood biomarkers could be useful, surpassing CSF proteomic biomarkers and detecting disease in earlier stages. These studies also provide glimpses for novel therapeutic targets toward the already established Aβ accumulation and tau hyperphosphorylation, immune-inflammatory responses, oxidative stress, energy and mitochondrial metabolism, synaptic plasticity, vesicle-mediated transport, lipid metabolism, and microvascular homeostasis (53). Using a non-targeted analysis of plasma proteins and machine learning, Ashton et al. (54) achieved good performance in a model that predicted Aβ in cognitively unimpaired individuals, while highlighting novel candidates for AD pathology among the 12 features utilized in the model. Chen and Xia (55) compared proteomic data from plasma and brain tissue in AD patients and in healthy controls, showing that the complement coagulation cascade and interleukin-6 signaling molecules are linked to AD, and proposing synchronic immune responses between the tissues in a chronic inflammatory state.

Beyond CSF and blood, other fluids have also been explored. Contini et al. (56) analyzed saliva and found that AD individuals presented increased expression of proteins involved in homeostasis, ROS scavenging, neuroprotection, and antimicrobial activity compared to controls, suggesting that the oral cavity of such patients also establishes a defensive state. In urine, by contrast, proteins related to lipoprotein metabolism, complement activation, and gluconeogenesis were altered (57), while in tear fluid, the eukaryotic translation initiation factor 4E was present only in AD samples (58). This factor has already been found to be increased in brain tissues of AD patients (59), and might be involved in the mechanisms behind tau hyperphosphorylation (60).

## Parkinson's Disease

Parkinson's disease (PD) is the second most prevalent neurodegenerative disease after AD. Recent reports indicate that both the incidence and prevalence of PD have increased significantly over the last two decades, making it one of the fastest-growing neurological disorders globally (61). PD presents a marked pathophysiological heterogeneous condition without reliable biomarkers to propose a biological definition of the disease, therefore its diagnosis relies mainly on clinical assessment. The most accepted mechanisms for disease onset involve α-synuclein misfolding and protein degradation impairment, including dysfunctions in the ubiquitin-proteasome and lysosomal autophagy pathways. These changes lead to aggregation of α-synuclein, the main component of Lewy bodies and Lewy neurites, histological hallmarks of the disease. Additionally, mitochondrial dysfunction and microglial-induced inflammatory responses are also implicated in PD neurodegeneration. Despite this knowledge, the diagnosis in earlier phases, prognostic markers and disease-modifying treatments are still missing, and metabolomics and proteomics studies hold promise for progress in these areas (62).

According to the current diagnostic criteria, PD is diagnosed when patients exhibit the classical motor features of bradykinesia, rest tremor and rigidity, but at this time, they already present 80% of loss of striatal dopamine (63). Accumulating evidence suggests that there is a prodromal phase before PD motor signs appear (5–15 years earlier), which represents an opportunity for earlier diagnosis with biomarkers and a window for disease-modifying treatments (64). Clinical features of this prodromal phase have been described, such as hyposmia, REM sleep behavior disorders (RBD), constipation, and depression, but they are unspecific. In this scenario, dopaminergic PET/SPECT imaging can detect preclinical dopamine dysfunction several years before disease manifestation in individuals at high risk, including those with RBD or a pathogenic dominantly inherited mutation (65–67).

### Metabolomics

PD is a disorder in which several genetic, environmental, and lifestyle factors play a role, making the study of multi-omics a promising approach to depict its heterogeneity. There is growing literature suggesting that a specific metabolic signature could differentiate patients according to their genetic status. Recently, Lerche et al. (68) combined the analysis of multiple metabolites in PD patients with and without *GBA* mutations and showed specific differential metabolite profiles regarding α-synuclein, glucocerebrosidase activity, and glucosylceramides between the two groups (40). These results suggest that *GBA* mutations greatly influence disease pathophysiology and exhibit a distinctive biomarker profile. Another recent research evaluated PD patients and unaffected controls with or without the *LRRK2* G2019S mutation and evidenced a differential metabolic profile between PD and controls, highlighting lower hypoxanthine and metabolites of purine pathway (69). Crotty et al. (70) studied how metabolomics is affected by *LRRK2* mutation status and performed plasma and CSF analyses in four groups of subjects: idiopathic PD, PD with a *LRRK2* mutation, unaffected controls, and unaffected controls with a *LRRK2* mutation. Interestingly, they reported caffeine and its metabolites to be decreased among PD patients, with higher levels in those carrying a *LRRK2* mutation, suggesting a protective effect of caffeine and an environmental interaction in genetic mutation carriers.

Another encouraging approach to uncover potential biomarkers or biochemical pathways contributing to disease is the investigation of candidate specific metabolic profiles in patients. Chang et al. (71) focused on profiling molecules involved in the kynurenine pathway of tryptophan metabolism, assessing the role of markers involved in oxidative stress and excitotoxicity such as plasma kynurenic acid, quinolinic acid, and kynurenine in PD onset and severity. An additional metabolic pathway profiled was that of the polyamines, where Saiki et al. (72) showed that spermine and N1,N8-diacetylspermine was also altered in PD patients, with increased levels consistent with disease severity. In a recent study, Shao et al. (73) performed a comprehensive unbiased metabolic profile using a liquid chromatography-mass spectrometry approach in 223 PD patients and 169 controls. The authors reported an extensive list of amino acids, acylcarnitines, organic acids, steroids, amides, and lipids from human plasma that might help unveil PD physiopathology and screen for potential therapeutic targets.

Serum uric acid (SUA) is one of the most studied metabolites in blood matrices. It has been associated with morbidity, severity, progression, non-motor symptoms and risk of developing PD (74) Uric acid (UA) levels are inversely correlated with development and progression of PD (75). This has been attributed to a possible neuroprotective role (76) and the suggested mechanisms implicated in it are: scavenging of free radicals, iron chelation, modification of genetic variability and countering apoptosis (75). Recently, van Wamelen et al. (77) observed a negative association between SUA and non-motor symptoms. Songsomboon et al. (78) proposed the use of SUA/serum creatinine as a more sensitive diagnostic tool, and Bougea et al. (79) suggested that SUA might be a marker specific for PD patients with a causative *LRRK2* mutation. In an umbrella review of risk factors and biomarkers for neurological diseases, Mentis et al. (80) confirmed that lower SUA levels may be associated with an increased risk of PD. Even so, UA might be acting as a confounder not eliciting a causative effect. Studies looking at the effect of lowering or raising UA levels have not demonstrated an association or benefit in PD patients. Lai et al. (81) conducted a case control study to determine the association between allopurinol, a gout treatment that reduces UA levels, and PD. Their results were negative, and concluded that there is no association between allopurinol treatment and PD (81). These findings are related to those published in a recent randomized clinical trial (66) that was unsuccessful in slowing early PD progression with inosine (urate precursor) and had to be interrupted.

An additional largely studied fluid for metabolomic studies in PD is CSF, which has shown interesting results. In a meta-analysis by Adani et al. (82), PD patients displayed higher levels of copper, iron and selenium in CSF without showing increased levels of these trace elements in peripheral blood matrices. This study suggests that PD promotes alterations in metal transporters or in the integrity of the blood-brain barrier (82). Interestingly, ceruloplasmin serum levels, a ferroxidase enzyme involved in copper and iron metabolism, have been linked to specific phenotypic PD features, such as impulsivity (82).

Along with metals, catecholamines metabolism in the central nervous system (CNS) of PD patients seems to be altered. Goldstein et al. (83) found differential profiles in CSF catecholamines between PD, multiple systems atrophy and pure autonomic failure, with a more prominent difference in markers of central dopaminergic deficiency. D'Andrea et al. (84) performed ultra-performance chromatography mass spectrometry analyses of plasma in 21 drug-naive *de novo* PD patients and suggested tyramine as a putative marker of early-stage PD, as well as suggested that tyramine, norepinephrine and tyrosine together may act as a prognostic marker. Wichit et al. (85) performed a high-performance liquid chromatography study of monoamines in the plasma of PD patients and identified a significantly higher homovanillic acid/dopamine ratio and a lower 5-hydroxyindoleacetic acid/serotonin ratio, emphasizing the involvement of multiple neurotransmission systems in the disease.

To a lesser extent, brain and urine sample studies have been conducted. In a meta-analysis of copper and iron, Genoud et al. (86) showed reduced copper and increased iron levels in postmortem substantia nigra of PD patients, suggesting further investigation of such metabolites as disease modification targets. Utilizing iPSC from *LRRK2* G2019S PD patients, Sonnien et al. (87) observed increased production of α-synuclein, altered metabolism and calcium homeostasis, increased release of cytokines, increased levels of polyamines and their precursors, and decreased levels of lysophosphatidylethanolamine in the astrocytes of *LRRK2* mutation PD patients. These findings suggest that astrocytes are likely to contribute to the pathogenesis of PD. Furthermore, Kumari et al. (88) observed increased levels of ornithine, phenylalanine, isoleucine, β-hydroxybutyrate, tyrosine and succinate in urine of PD patients using nuclear magnetic resonance, showing disturbances in multiple metabolic pathways. The authors propose the use of such metabolites as complementary diagnostic markers, considering that urine is a non-invasive matrix (88).

The gut microbiome has been increasingly recognized as a critical factor in PD pathophysiology. Tan et al. (89) analyzed the fecal microbiome and metabolome of 104 patients and 96 controls and found changes in the levels of metabolites with putative neuroprotective effects, such as short chain fatty acids, ubiquinones, and salicylate, along with other compounds previously related to neurodegeneration, such as ceramides, sphingosine, and trimethylamine N-oxide. Notably, Hertel et al. (90) performed a multi-omics longitudinal analysis of 30 *de novo* PD patients and 30 controls, and observed longitudinal alterations in methionine and cysteine levels, along with differences in taurine-conjugated bile acids and sulfated taurolithocholate. This study suggests different research routes for understanding the disease, specially at the sulfur host-microbial interactions and, consequently, bile-acid metabolism. Unsurprisingly, Vascellari et al. (91) also associated microbiota composition with metabolomic analyses, and suggested synergistic relationships between gut microbes and bacterial metabolites in association with PD.

### Proteomics

With a less expressive body of knowledge than that of AD, proteomic approaches in PD have been progressively explored by researchers. While not fully explaining disease development, many protein studies focus on biomarkers for diagnosis and prognosis of patients, such as α-synuclein (92), neurofilament light chain protein (NfL) (93), or even amyloid beta and phosphorylated tau in addition to other proteins commonly associated to AD (94).

A systematic review and meta-analysis showed that total α-synuclein was reduced in CSF of PD patients compared to controls, while oligomeric and phosphorylated α-synuclein were increased (95). Sensitivity and specificity for differentiating PD from controls were 0.72 and 0.65 for total α-synuclein and 0.71 and 0.64 for oligomeric α-synuclein, respectively. Later in 2019, a longitudinal analysis of α-synuclein levels in the CSF of patients with prodromal and early PD revealed that baseline α-synuclein was lower in patients with manifest and prodromal PD compared to healthy controls (96). At 24 and 36 months, α-synuclein levels declined considerably in PD, remained unchanged in prodromal PD, and trended upward in healthy controls. The authors concluded that, whereas CSF α-synuclein is related to PD, it does not correlate with disease progression and hence does not indicate ongoing dopaminergic deterioration.

With broader understanding of the pathways surrounding protein alterations, proteomic approaches have raised important novel molecules to PD development theories. Using liquid chromatography-tandem mass spectrometry to quantify 341 groups of proteins in the CSF of PD patients and controls, Rotunno et al. (97) identified several altered proteins and protein ratios. Specific attention was given to significant reduction in proteins of the granin family, supporting larger catecholaminergic alterations in PD. Raghunathan et al. (98) found proteomic changes in Brodmann area 9 of PD brain tissue including collagens, proteoglycans, and hemoglobin chains, the latter ones suggesting defects in iron metabolism. In a systematic review and meta-analysis, Monti et al. (99) explored data from proteomic studies of neurodegenerative diseases and performed a network analysis, raising important differences in pathways involved in neuronal death and loss of specific neuronal populations in PD patients.

Additionally, Virreira Winter et al. (100) found specific proteomic profiles in the urine of PD patients. Examining protein levels through a machine learning algorithm, the authors discriminated *LRRK2* G2019S mutation status and disease manifestation in mutation carriers with an AUC of 0.84. As an alternative matrix, Boerger et al. (101) explored proteins in tear fluid of PD patients, revealing altered proteins involved in oxidative stress, immune response, and lipids metabolism. Major alterations in lipids metabolism were also found by Hu et al. (102) in a proteomic and metabolomic analysis of fasting plasma from PD patients. In this study, out of the forty differentially expressed proteins, seven were apolipoproteins (102).

Further interest was also raised around protein content of exosomes and nanometer-sized vesicles. Jiang et al. (103) performed mass spectrometry analyses of label-free serum exosome proteins, identifying differential expression in 14 proteins, including complement C1q, apolipoprotein D, pigment epithelium-derived factor, and gelsolin. In previous work, Kitamura et al. (104) utilized two-dimensional differential gel electrophoresis and found alterations in levels of clusterin, complement C1r, apolipoprotein A1, and fibrinogen gamma chain in extracellular vesicles from plasma samples of PD patients.

A recent study (67) suggested that both plasma and CSF levels of NfL could be a useful prognostic biomarker for PD (67). The study included 152 PD patients, and showed that NfL levels in plasma and CSF predicted change in cross-sectional associations between NfL and the Unified Parkinson's Disease Rating Scale Part III (UPDRS-III) and Mattis Dementia Rating Scale (DRS-2) scores using linear mixed-effects models. This article suggests that PD individuals with plasma NfL values in the highest tertile were five times more likely to convert to MCI or dementia during follow-up.

## Amyotrophic Lateral Sclerosis

Primarily affecting the upper and lower motor neurons, ALS is a dramatically progressive and incurable neurodegenerative disease (105). To date, the identification of biomarkers has been less elusive to identify etiological pathways in ALS than in AD and PD. With an increasing prevalence worldwide, a massive number of individuals will continuously struggle to survive this chronic, but severely debilitating disease. Hence, it is essential that we work to understand the mechanisms of this devastating disease in order to identify biomarkers and points of intervention. The “omics” approach may provide unique targets to develop reliable diagnostic markers.

Currently, the diagnosis of ALS is based on clinical symptoms and electroneuromyography studies (105). A myriad of different phenotypes have been identified in the spectrum of ALS, which involves distinct neuronal topographies, clinical extension and rate of progression (106). Bulbar, limb-onset and signs of frontotemporal dementia are the most frequently described subtypes in ALS with varying progression rate (107). A large body of research prevails, but a minor number of biomarkers have been validated accurately (108). Even though studies have failed at identifying hallmarks of ALS, the future holds promise (109). For instance, it is well-established that individuals with ALS present pathological hypermetabolism (110, 111), leading to early cell dysfunction and worse prognosis.

While fluid biomarkers including plasma and CSF, are still unavailable in the clinical setting, current efforts are in uncovering the etiological basis of ALS with different approaches. ALS-linked genetic mutations have been identified in familial, but also sporadic ALS (112), and they may indicate specific pathological pathways. A novel approach using induced iPSC derived from patients with ALS has opened opportunities to study ALS *in vitro* as reviewed elseqhere (112). Integration of multimodal data with machine learning has also a potential to change paradigms in the ALS pathway discovery (113). Furthermore, the *omics* field presents numerous advantages at identifying pathological networks in comparison with other disciplines. The next section details state-of-the-art knowledge of metabolomics and proteomics studies aimed at uncovering the mist around biomarkers of ALS.

### Metabolomics

The identification of pathway specific alterations has thus far helped uncover several molecules implicated in ALS, and suggests a key role for aberrant mechanisms in ALS pathogenesis. By measuring metabolites such as glutamate, antioxidants, and lipids, we have gained insights into mechanisms of glutamatergic excitotoxicity, oxidative stress, and mitochondrial dysfunction involved in disease etiology (114). Metabolomic studies have enabled the discovery of a myriad of substances that may be involved in the etiology of ALS, with the hope that in the future biomarkers for diagnostics, prognostic and disease progression may be possible in clinical practice.

Untargeted metabolomics performed on the plasma of ALS patients and controls have highlighted many known players in ALS etiology, such as sphingolipids, which are involved in autophagy and inflammation (115). However, it has also identified abnormalities in novel pathways, such as benzoate metabolism, which the authors suggest may be explained by pesticide exposure, and diacylglycerols, which have roles in inflammation, immune cell signaling, and apoptosis (115). Carbohydrates have shown contradictory results, though their role is still to be uncovered (108).

Lipid metabolite imbalance has been identified in patients with ALS with promising results. A recent study found a panel of cholesteryl esters, di- and triglycerides among other lipids to be associated with ALS pathological pathways (116). A 20-year follow-up study identified that serum low-density and high-density lipoprotein cholesterol (LDL-C and HDL-C), apolipoprotein B and other lipids were associated with increased incidence of ALS (117, 118). Plasma and CSF levels of lipids were correlated with disease progression (119), providing insights into predicting models for worse prognosis. A CSF lipid signature is also associated with survival rates (120). Dyslipidemia is also pointed out as part of the etiological mechanisms involved on ALS, opening the possibility for implementation of nutritional changes (121).

As seen in other neurodegenerative diseases, metabolomics holds significant promise in discovering biological pathways associated with genetic mutations (122). ALS-linked genetic mutations such as in *TARDBP*, encoding TDP-43, lead to decreased carnitine, increased pyruvate and fatty acids (112, 123). Besides, ALS individuals with a *C9orf72* mutation present lower HDL-C when compared with ALS non-carriers (112). *SOD1* mutations in ALS have also demonstrated a distinct metabolic phenotype, such as a decrease in arginine, lysine, ornithine, serine, threonine and pyroglutamic acid, in patients carrying a D90A SOD1 (122). Additionally, research studies have suggested potential for intervention in pathways highlighted by metabolomics studies (124, 125) highlighting that alterations in pathways may be associated with risk of ALS before diagnosis (126). Importantly, though, not all patients may present with the same metabolic abnormalities (127), reinforcing the need for determination of compatible subgroups and personalized medicine.

### Proteomics

Several proteins have been implicated in both familial and sporadic ALS, such as TDP-43 (128) and SOD1 (129), among others. Whether these inclusions are a symptom or a cause of cellular degeneration in ALS remains to be elucidated, but recent advances in proteomics have greatly improved our understanding of the pathways and mechanisms in which these proteins are involved.

The protein degradation pathway has been heavily implicated in ALS pathogenesis, specifically with regard to the aforementioned inclusions (130). A mutation in *CCNF*, the gene encoding cyclin F, found in familial and sporadic ALS patients, has been examined in Neuro-2A and SH-SY5Y cells. This study revealed defects in the autophagy pathway impairing autophagosomal-lysosome fusion (131), and potentially a mechanism for the accumulation of substrates such as TDP43 (132) and SOD1 (133). *C9orf72* has also been implicated in the autophagy pathway; specifically through an association with ULK1-type complexes (134, 135) via stable isotope labeling by amino acids in cell culture (SILAC). Traditional SILAC, though, is only capable of cell protein isotope labeling and quantification, not real-time or *in vivo* measurements, a potential limitation going forward.

An analysis of the CSF proteome of ALS patients revealed a panel of candidate biomarkers implicated in synaptic activity, inflammation, glial response, axonal damage and apoptosis (114, 136, 137). Label-free LC-MS analysis showed NfL, and C3 secretogranin pathways with high sensitivity and specificity in distinguishing ALS from controls (138). Many other proteins have also shown promising results, though further studies are needed to confirm these results, including CHIT1, GRIA4, and Cystatin C (139, 140). Proteomic analysis of CSF-derived extracellular vesicles, which aims to provide novel insights into key processes associated with ALS pathogenesis, showed downregulation of proteasome core complex proteins through gene ontology enrichment analysis (141).

The axonal cytoskeletal protein NfL and its phosphorylated form pNFH are amongst the most studied proteins associated with ALS (142). NfL has been validated as a diagnostic biomarker in plasma for ALS (143), particularly due to its high accuracy in differentiating ALS to ALS-mimics phenotypes (144). NfL has demonstrated different levels in distinct clinical phenotypes, providing key insights into the biological meaning of this protein (145). Both NfL and pNFH have been shown to be correlated to shorter life expectancy in patients with ALS, more rapid progression (145, 146) and generally worse prognosis. It is also feasible to measure these proteins in plasma (147), which increases applicability in clinical practice.

## Conclusions

The advent of high-throughput platforms for the detection of proteins and metabolites followed by the implementation of multimodal approaches may shed some light on the biochemical signatures involved in disease etiology. Research performed in the proteomics and metabolomics space including large-scale longitudinally followed and well-characterized case cohorts will facilitate the identification of biomarkers for prediction, diagnosis and prognosis of neurodegenerative diseases. It is our hope that the integration of these data modalities will put the field on a path toward better therapeutics that target the most appropriate patients early in their disease course with effective interventions.

Though promising, metabolomics and proteomics are still in its early days, with many challenges to overcome. One of the major caveats is the lack of standardization between studies, which is particularly challenging in measuring small substances. Current research has shown panels of metabolites and proteins that were not replicable, or partially replicable with almost no overlap across studies (148). To harmonize measures and mitigate the inter-study heterogeneity, a standardized operational procedure (SOP) should be applied (148). The ideal path forward would be integration of these various analyses through the prioritization of multi-omics. For instance, a multi-omic analysis that includes transcriptomic, metabolomics, and proteomic data could be used to understand pathways of selective vulnerability. Additionally, the increasing prevalence of single cell metabolomics and proteomics will undoubtedly help to further elucidate the nuances of neurodegenerative diseases.

## Author Contributions

All authors listed have made a substantial, direct, and intellectual contribution to the work and approved it for publication.

## Funding

This research was supported, in part, by the Intramural Research Program of the National Institutes of Health (National Institute on Aging, National Institute of Neurological Disorders and Stroke: project numbers 1ZIA-NS003154, Z01-AG000949-02, and Z01-ES10198).

## Conflict of Interest

The authors declare that the research was conducted in the absence of any commercial or financial relationships that could be construed as a potential conflict of interest.

## Publisher's Note

All claims expressed in this article are solely those of the authors and do not necessarily represent those of their affiliated organizations, or those of the publisher, the editors and the reviewers. Any product that may be evaluated in this article, or claim that may be made by its manufacturer, is not guaranteed or endorsed by the publisher.
